# Patient Preferences for Receiving Education on Venous Thromboembolism Prevention – A Survey of Stakeholder Organizations

**DOI:** 10.1371/journal.pone.0152084

**Published:** 2016-03-31

**Authors:** Victor O. Popoola, Brandyn D. Lau, Hasan M. Shihab, Norma E. Farrow, Dauryne L. Shaffer, Deborah B. Hobson, Susan V. Kulik, Paul D. Zaruba, Kenneth M. Shermock, Peggy S. Kraus, Peter J. Pronovost, Michael B. Streiff, Elliott R. Haut

**Affiliations:** 1 Department of Surgery, The Johns Hopkins University School of Medicine, Baltimore, Maryland, United States of America; 2 Department of Anesthesiology and Critical Care Medicine, The Johns Hopkins University School of Medicine, Baltimore, Maryland, United States of America; 3 Department of Medicine, The Johns Hopkins University School of Medicine, Baltimore, Maryland, United States of America; 4 Division of Health Sciences Informatics, The Johns Hopkins University School of Medicine, Baltimore, Maryland, United States of America; 5 Department of Emergency Medicine, The Johns Hopkins University School of Medicine, Baltimore, Maryland, United States of America; 6 Department of Nursing, The Johns Hopkins Hospital, Baltimore, Maryland, United States of America; 7 Department of Pharmacy, The Johns Hopkins Hospital, Baltimore, Maryland, United States of America; 8 The Armstrong Institute for Patient Safety and Quality, Johns Hopkins Medicine, Baltimore, Maryland, United States of America; 9 Department of Health Policy and Management, The Johns Hopkins Bloomberg School of Public Health, Baltimore, Maryland, United States of America; Ottawa Hospital Research Institute, CANADA

## Abstract

**Importance:**

Venous thromboembolism (VTE) is a major cause of morbidity and mortality among hospitalized patients and is largely preventable. Strategies to decrease the burden of VTE have focused on improving clinicians’ prescribing of prophylaxis with relatively less emphasis on patient education.

**Objective:**

To develop a patient-centered approach to education of patients and their families on VTE: including importance, risk factors, and benefit/harm of VTE prophylaxis in hospital settings.

**Design, Setting and Participants:**

The objective of this study was to develop a patient-centered approach to education of patients and their families on VTE: including importance, risk factors, and benefit/harm of VTE prophylaxis in hospital settings. We implemented a three-phase, web-based survey (SurveyMonkey) between March 2014 and September 2014 and analyzed survey data using descriptive statistics. Four hundred twenty one members of several national stakeholder organizations and a single local patient and family advisory board were invited to participate via email. We assessed participants’ preferences for VTE education topics and methods of delivery.

Participants wanted to learn about VTE symptoms, risk factors, prevention, and complications in a context that emphasized harm. Although participants were willing to learn using a variety of methods, most preferred to receive education in the context of a doctor-patient encounter. The next most common preferences were for video and paper educational materials.

**Conclusions:**

Patients want to learn about the harm associated with VTE through a variety of methods. Efforts to improve VTE prophylaxis and decrease preventable harm from VTE should target the entire continuum of care and a variety of stakeholders including patients and their families.

## Introduction

Venous thromboembolism (VTE), which comprises deep vein thrombosis (DVT) and pulmonary embolism (PE), is one of the most common causes of preventable harm among hospitalized patients [1–6]. The Agency for Healthcare Research and Quality (AHRQ) has endorsed practices to prevent VTE as a top patient safety strategy in hospitals [7–10]. Consequently, numerous interventions to improve VTE prevention have focused on improving prescription of appropriate prophylaxis [11]. This strategy is necessary though not sufficient: hospitalized patients do not always receive the medications they are prescribed.

Previous studies have shown that a substantial number of doses of prescribed pharmacologic VTE prophylaxis are not administered to patients [12] and that the most frequently documented reason for non-administration is patient refusal [13]. Medically-ill (compared to surgical) patients were most likely to refuse medication doses and half of all patients missed one or more of their prescribed doses [12]. These missed doses are associated with VTE events and preventable patient harm [14, 15].

The National Blood Clot Alliance (NBCA) surveyed 500 patients with a recent history of hospitalization, and found a significant gap in participants’ knowledge of VTE [16]. Among respondents, 28% and 15% had basic knowledge of DVT or PE, respectively, yet 15% of the participants had a personal history and 43% had a family history of DVT or PE. More recently, and as one of the activities of World Thrombosis Day (October 13, 2014), Wendelboe and colleagues carried out a global survey among 7233 participants in nine countries, to determine the level of awareness of VTE including risk factors, signs and symptoms [17]. They found awareness to be lowest for DVT (44%) and PE (54%) compared to other common conditions such as breast cancer (85%), stroke (85%), prostate cancer (82%), and heart attack (88%).

Multiple factors operating at different levels of care influence whether or not a patient receives appropriate VTE prophylaxis. Numerous interventions target the prescribing clinician [11]. However, nursing beliefs regarding VTE risk and pharmacologic prophylaxis also influence administration of appropriately prescribed medication [18]. Given the high incidence of documented patient refusal and identified knowledge gaps among patients at-risk for VTE, patients and their family members, if educated and engaged in their care, could be a potent intervention in preventing VTE. Patients should be educated and empowered to be active partners in making informed decisions regarding their own health.

The purpose of this study was to develop a patient-centered approach to educating patients and their family members on the harms of VTE and importance of VTE prophylaxis in hospital settings. Using a modified Delphi method, we engaged members of three national stakeholder organizations focused on VTE prevention and treatment and a local, hospital-based, patient advisory council, to build consensus on the content and approaches to delivery of information related to VTE prevention in hospitalized patients.

## Materials and Methods

From March 2014 to September 2014, we engaged a national sample of patients and family members on the content and approaches to delivery of information related to VTE prevention in hospitalized patients. To build consensus, we employed a modified Delphi approach, an iterative process of obtaining input from experts and working towards consensus [19]. Members of the North American Thrombosis Forum (NATF), the National Blood Clot Alliance (NBCA), Clot Care, and The Johns Hopkins Hospital Patient and Family Advisory Council were invited to participate. Participants were recruited via email and/or social media (websites, Facebook, Twitter) through their respective organizations and their responses were collected using an interactive, three-phase, web-based survey tool (SurveyMonkey, Palo Alto, CA). Informed consent was obtained from participants and email addresses were collected to link multiple surveys.

During Phase 1, respondents were asked to self-report demographic characteristics including age, race, gender, highest education attained, healthcare-related training or experience, and personal or family history of VTE. In Phase 2, respondents were asked to identify broad areas of patient-clinician communication needs and approaches to delivery. For each question, a field was included for free text so that respondents could volunteer additional information they considered important. Based on feedback from Phase 2, respondents were asked to rank their top four educational topics (from first through fourth choice) and top three methods of delivery (from first through third choice). Preference ranks for educational topics were assigned weights, the highest weight of 4 was assigned to the first choice category and the lowest weight of 1 was assigned to the fourth choice category. Participants were required to rank all four educational topics. Similarly, preference ranks for the approaches to delivery of VTE education were assigned weights from a maximum weight of 3 for the first choice category to a weight of 1 for the third choice category. An unranked category had a weight of 0. In addition, respondents were asked to identify the maximum amount of time they would be willing to spend reviewing educational materials as hospitalized patients.

For Phase 2, we defined consensus as a simple majority of respondents. To determine consensus in Phase 3, we employed the Borda Count, a well-recognized statistical approach for aggregating rankings. This consensus based voting system accounts for both popularity and preference [20, 21]. The weights associated with participants’ preference selections were summed in each category to produce the Borda Count for that category. Mean weights were calculated as a simple average of weights allocated to the categories by all respondents. Categories were compared using the Kruskal-Wallis rank sum test. Data was analyzed using Stata SE (version 13.1; Stata Corp.) and Microsoft Excel 2013 (Microsoft). This research study which involves a survey procedure with minimal risk to participants was approved by the Johns Hopkins Institutional Review Board (NA_00091152) with an exemption.

## Results

### Phase 1

During the Phase 1 assessment, 421 individuals were recruited through our three national partnering stakeholder organizations and the local patient advisory committee. Of the 421 respondents, 251 (59.6%) were recruited from the NBCA, 156 (37.0%) from Clot Care, 7 (1.7%) each from the NATF and The Johns Hopkins Hospital Patient and Family Advisory Council. Respondents were disproportionately female (331/421, 78.6%) and white (376/421, 89.3%) (See Table 1). The median age of respondents was 47 years (IQR: 37–58; range: 17–82). The majority of respondents lived in the United States (354/421, 84.1%) and had earned at least a college degree (373/421, 88.6%). One-third (139/421, 33.0%) of respondents had any form of medical or healthcare training. Most respondents had a personal history of VTE (330/421, 78.4%) and more than one-third (169/421, 40.1%) had a family history of VTE. The majority (137/169, 81.1%) of respondents who had a family history of VTE also had a personal history of VTE.

**Table 1:** Demographic characteristics of respondents.

Characteristics	Frequency (%) N = 421
**Age** [Median (IQR)]	47.0 (37–58)
**Gender**	
Female	331 (78.6)
Male	88 (20.9)
Prefer not to answer	2 (0.5)
**Race**	
American Indian or Alaska Native	2 (0.5)
Asian	8 (1.9)
Black or African American	17 (4.0)
White	376 (89.3)
Other (please specify)	13 (3.1)
Prefer not to answer	5 (1.2)
**Level of Education**	
College degree or higher	323 (76.7)
No College Degree	94 (22.3)
Prefer not to answer	4 (1.0)
**Healthcare Training**	
Yes	139 (33.0)
No	281 (66.7)
Prefer not to answer	1 (0.2)

### Phase 2

The purpose of the Phase 2 survey was to identify education topics and approaches to delivery for engaging and educating hospitalized patients on VTE prevention. The Phase 2 survey was sent to all 421 respondents in Phase 1, of which 227 (53.9%) responded. Of the 227 respondents, 186 (81.9%) wanted to talk to a physician, 145 (63.9%) wanted to receive information about VTE on a piece of paper, 105 (46.3%) wanted to talk to a nurse, and 41 (18.1%) wanted to talk to a pharmacist. Ninety-nine (43.6%) respondents wanted to watch a video on a smartphone or television. Asked separately whether or not they would be willing to read a piece of paper if this was the only available medium, 88.1% (200/227) responded affirmatively.

To identify patient preferences for route of administration of VTE prophylaxis, we asked respondents for their preferred route of administration if a shot and an equally effective oral medication for VTE prophylaxis were available. Of 227 respondents, 178 (78.4%) preferred pills, 31 (13.7%) preferred shots, and 18 (7.9%) had no preference (See Table 2).

**Table 2:** Preferred route of administration of Pharmacologic VTE prophylaxis.

Preferred Route of VTE Prophylaxis Administration	Frequency (%) N = 227
Pill	178 (78.4)
Shot	12 (5.3)
No preference	31 (13.7)
No response	6 (2.6)

To obtain consensus on the most effective overarching message regarding VTE, we asked respondents to describe their preferred context for receiving information on VTE. Nearly half of all respondents (107/227, 47.1%) wanted information that would emphasize the danger of VTE especially relative to other common conditions. Less than a third (66/227, 29.1%) wanted information that emphasized the preventable nature of VTE.

### Phase 3

The Phase 3 survey was sent to all 421 respondents from Phase 1, from the four stakeholder organizations. We received responses from 215 respondents (51.1%). Respondents were asked to rank their preferences among the presented options of education content and approaches to delivery identified in Phase 2. Of 215 respondents and without regards to preference rank, 181 (84.2%) would like to receive education about VTE by talking to a physician, 140 (65.1%) wanted to watch an educational video on a TV, tablet or smartphone, 136 (63.3%) wanted to read from a piece of paper, 118 (54.9%) would like to receive education by talking to a nurse, and 43 (20%) wanted to talk to a pharmacist. Most patients (141, 65.6%) selected the option for talking to a physician as their most preferred method of receiving education on VTE; only 32 (14.9%) selected a video as their most preferred method while 29 (13.5%) wanted to learn about VTE by reading a piece of paper as their top choice. Only 6 (2.8%) respondents selected talking to a nurse or talking to a pharmacist as their most preferred method.

**Fig 1:**
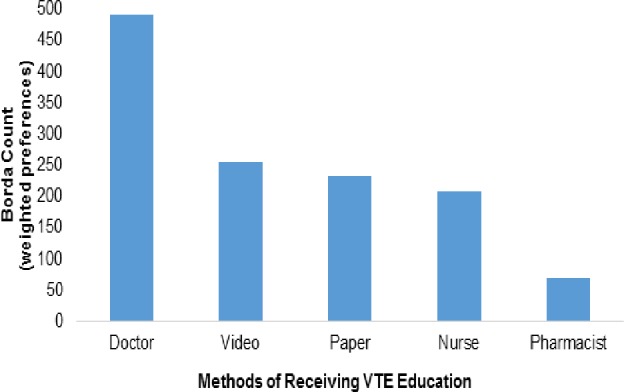
Respondents’ preferences for receiving VTE education (methods). A bar graph showing stakeholders’ preferred methods for receiving VTE education. Respondents were asked to rank methods of receiving VTE education in order of preference. Preference categories, 1^st^, 2^nd^ and 3^rd^ were assigned weights of 3, 2 and 1 respectively and a category that is not ranked was assigned a weight of 0. Borda Counts were derived as the sum of weights allocated to the respective preference ranks by participants.

To determine the methods with the highest priority while also accounting for popularity, we compared the Borda Count for each category. The most-preferred methods for receiving education about VTE (Borda Counts, mean weights) were, talking with a doctor (491, 2.3), watching a video on a TV, tablet or smartphone (255, 1.2), reading from a piece of paper (232, 1.1) and talking with a nurse (209, 1.0). There was a statistically significant difference (p<0.0001) between the ranks allocated to those methods (See Fig 1).

**Fig 2:**
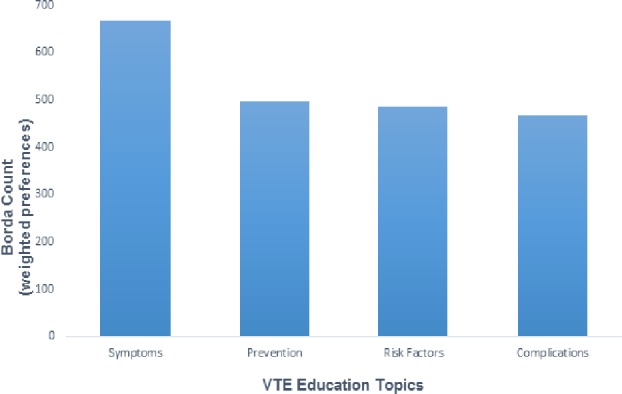
Respondents’ preferences for receiving VTE education (topics). A bar graph showing stakeholders’ preferred VTE education topics. Patients were asked to rank VTE education topics in order of preference. Preference categories, 1st, 2^nd^, 3rd and 4^th^ were assigned weights of 4, 3, 2 and 1 respectively. Borda Counts were derived from the aggregated products of frequency and weights of the respective preference categories.

When asked what they would prefer to learn the most about VTE, nearly half of respondents (100 of 212, 47.2%) were most interested in learning how to recognize signs and symptoms, and of the remaining 112 respondents, 58 (51.8%) selected this as their second most-preferred topic of interest. Thirty-nine respondents (18.4%) were most interested in learning how to prevent VTE, 38 (17.9%) were most interested in learning their risk for having a blood clot and 35 (16.5%) were most interested in learning the possible consequences of a blood clot. Overall, there was a statistically significant difference between the ranks allocated to the various topics (p<0.0001). The item receiving the highest Borda Count was “symptoms” (Borda Count = 668, mean weight = 3.2). The other 3 items (“prevention”, “consequences” and “risk”) were very similar with Borda Counts and mean weights of 498;2.3, 486;2.3 and 468;2.2, respectively (p = 0.3) (See Fig 2).

The vast majority of respondents were willing to read a 1-page (199, 93.9%) or 2-page (197, 92.9%) piece of paper. Smaller proportions were willing to read a 3-page handout (n = 156, 73.6%) or guidelines from national clinical organizations (n = 153, 72.2%).

**Fig 3:**
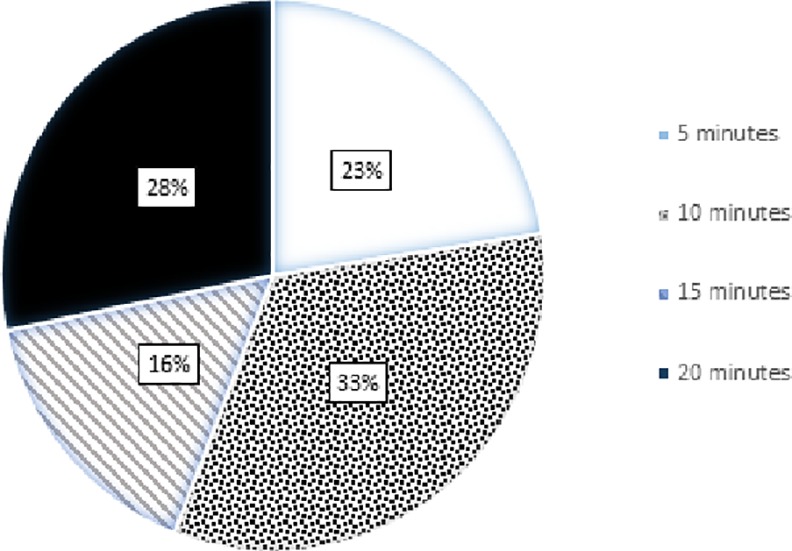
Respondents’ preferences for the length of educational material (video). A pie chart showing stakeholders’ preferences in regards to the length of educational material (video). Patients were asked whether or not they would be willing to watch a video that is 5, 10, 15 or 20 minutes long.

When asked what they would like to see in a video if this was the method of education, the overwhelming majority (181, 85.4%) preferred to see both patients and clinicians talking about VTE. All 212 respondents were willing to watch an educational video. The respondents had varying preferences for video duration; 110 (51.9%) were willing to watch 10 minutes or less of video while about half (102, 48.1%) were willing to watch a longer video of 15–20 minutes (See Fig 3).

## Discussion

Educating patients has been shown to improve understanding of the necessity of medication, reduce perception of harm from treatment and potentially prevent adverse clinical outcomes [22]. However, little has been written about the process by which the target audience is included in the planning and creation of healthcare educational materials. One strategy to improve compliance is engaging patients to make informed decisions about their preventive care by improving the quality of healthcare worker-patient communication [23, 24].

We found that most patients want to learn about a variety of VTE education topics from their physician supplemented by a variety of approaches including watching videos, talking with a nurse, and reading written materials. Although the majority of our respondents (81.9%) indicated that they would ideally prefer to receive education in the context of a patient-doctor encounter, a significant proportion of respondents also wanted to receive education by reading from a piece of paper (63.9%), talking to a nurse (46.3%), or watching a video (43.6%). Most respondents (65.6%) ranked talking to a physician as their most preferred method of education while less than a fifth each selected watching a video or reading from a piece of paper as their most preferred. Less than 3% selected talking to a nurse or talking to a pharmacist as their most preferred method of education. These findings suggest that patients’ perceptions of clinicians’ roles, whether real or imagined, may influence their health behavior.

Respondents were asked to select the VTE topic on which they would most like to be educated. The leading topic of choice was to learn about signs and symptoms of VTE, while the three remaining topics (prevention, risk factors, and consequences of VTE) were nearly equivalent. Most patients wanted to learn about VTE in a context of harm. This is not surprising as the perceived threat of a health condition is known to be an important determinant of patients’ health behavior [25]. However, previous studies of health behavior have found that while patients sometimes expect to be afraid when presented with their risk, most tend to demonstrate an “optimistic bias” or an “illusion of invulnerability” with regards to their personal risk [26, 27]. Hence, greater emphasis on risk in patient education may not necessarily result in a higher likelihood of adherence. In our study, more than 80% of respondents that had a family history of VTE also had a history of VTE. This suggests a significant gap in targeted VTE prevention efforts even among such high risk patients.

Education of health care professionals and clinical decision support within computerized provider order entry systems has been shown to result in improvements in VTE prophylaxis prescribing practices [28–32]. However, it is not enough to educate providers and improve their prescription behaviors regarding appropriate VTE prophylaxis regimen. We must also educate patients and their families on the importance and health benefits of adequate VTE prophylaxis and ensure better communication between clinicians and their patients so that patients can make the most informed decision regarding their care. All phases of VTE prevention are important in order to provide high-quality care. The acceptance of the medication by the patients is critical step along the way. We hope that by educating patients, we can decrease the rate of refusal of these medications and prevent more VTE [12, 15].

This study has important policy and practice implications and highlights significant gaps in educating and engaging patients. Although there is significant effort to leverage non-physician providers to educate patients, patients overwhelmingly prefer to receive education from their physicians. Although patients prefer to receive supplemental education through a variety of mechanisms such as videos, text, other types of clinicians, patient education is increasingly being linked to the electronic medical record that offers one mode of education.

Based on the results of this work, we have created the specific educational materials that patients and their families have asked for. We created a 2-page educational handout that is now the standard first-line educational material on VTE for all patients admitted to The Johns Hopkins Hospital. This material has been translated into multiple languages (including Spanish, Arabic, Chinese, Korean, Portuguese, Russian, and Nepali) and is available to all patients, their families, and the public via The Johns Hopkins VTE Collaborative website (http://www.Hopkinsmedicine.org/Armstrong/bloodclots). We also produced a 10-minute patient-centered, educational video with segments of four clinicians and six patients discussing the topics that the survey respondents requested; signs and symptoms, prevention, risk factors and consequences. This video is also being used at our hospital and can be viewed by the public as well (http://www.hopkinsmedicine.org/armstrong_institute/improvement_projects/VTE/patients.html)

Although not the primary objective of this project, it is important to remember that clinicians may not always know patient preferences for treatment and for education and we need to ask specifically. There are only a few patient populations in which oral regimens for VTE prophylaxis is indicated; this is a growing group with the assumption that ALL patients prefer an oral agent over an injectable medication. While it is true that three quarters of patients (78.4%) have this stated preference, some patients preferred shots (13.7%) or had no preference (7.9%). These numbers from this national survey are similar to a sample of inpatients at our hospital with a different (more representative) demographic breakdown that found only 60% of patients preferred an oral medication [33]. Similarly, some patients might prefer video over text.

Our study had a few limitations. The first relates to a potential selection bias: the majority of participants were female and white, and most had college degrees so our results may not be generalizable to other demographic strata; furthermore, only half of participants responded to the Phase 2 and Phase 3 surveys. Second, nearly 80% of respondents had a personal and/or family history of VTE. It is possible that preferences among patients who have never had a blood clot may be different than those surveyed. Third, we did not stratify our results by patient characteristics such as education, race, ethnicity or primary language and some types of patients preferences may differ from our findings. Fourth, we did not evaluate whether the education program improved patient education, engagement or outcomes. The scope of this paper was to describe the survey findings.

In conclusion, the results of this study suggest that patients want to be educated primarily by their physician supplemented with a variety of educational methods about VTE prevention. They want to understand how to recognize the signs and symptoms of VTE, their personal risk for VTE and the consequences of developing VTE. The hallmark of patient-centered care is that patients should be enabled to make informed decisions regarding their care. To do so, patients and their families must be educated and engaged. Hence, efforts at improving VTE prophylaxis and decreasing preventable harm from VTE should not be limited to health care workers but should target a variety of stakeholders including patients and their families. Clinicians can use this study’s findings to help educate and engage patients about VTE. Moving forward, we should solicit and use explicit patient preferences when creating educational materials geared toward patients and their families.

## Supporting Information

S1 File Participant Responses to Phase 2 Survey. (XLSX)

S2 File Participant Responses to Phase 3 Survey. (XLSX)

S3 File Survey Instruments. (DOCX)
