# Supplementary material for: Patient Preferences for Receiving Education on Venous Thromboembolism Prevention – A Survey of Stakeholder Organizations
Source: PLoS One. 2016 Mar 31;11(3):e0152084. doi: 10.1371/journal.pone.0152084 (PMC4816559; doi:10.1371/journal.pone.0152084)
Supplement: S3 File — (DOCX) [file pone.0152084.s003.docx]

**Patient Preferences for Receiving Education on Venous Thromboembolism Prevention – A Survey of Stakeholder Organizations**

**eMethods: Survey Instruments**

**Phase 1 - Demographics**

**What is your age? (years)**

**What is your ethnicity?**

Hispanic or Latino

Not Hispanic or Latino

Prefer not to answer

**What is your race?**

Black or African American

White

Asian

Pacific Islander/ Native Hawaiian

American Indian or Alaska Native

Prefer not to answer

Other (please specify)

**What is your gender?**

Female

Prefer not to answer

Other (please specify)

**What is the highest level of education you have completed?**

Did not attend school

Less than high school

Some high school

High school graduate or equivalent (GED)

Some college no degree

Graduated from college

Master’s degree

Advanced professional degree

Doctorate degree

**What is your total household income?**

Less than $25,000

$25,000 to $50,000

$50,001 to $75,000

$75,001 to $100,000

$100,001 to $150,000

More than $150,000

Prefer not to answer

**Where do you primarily live?**

In the United States

Outside of the United States

Prefer not to answer

**In what US state or territory do you live?**

**Do you have any medical/healthcare training?**

No

Yes (Physician)

Yes (Nurse)

Yes (Pharmacist)

Yes (Medical Assistant)

Yes (Physician Assistant or Nurse Practitioner)

Yes (Therapist [physical, respiratory, occupational, speech])

Yes (Technician [pharmacy, radiology, surgical])

Yes (Student [nursing, medical, pharmacy, student in any field above])

Yes (Other-please specify below)

Prefer not to answer

**Have you ever had a deep vein thrombosis/DVT (blood clot in the leg) or pulmonary embolism/PE (blood clot in the lung)?**

Yes

No

Unsure

**Has a family member ever had a deep vein thrombosis/DVT (blood clot in the leg) or pulmonary embolism/PE (blood clot in the lung)?**

Yes

No

Unsure

**Are you a member of the organizations below? Click all that apply**

National Blood Clot Alliance (NBCA)

North American Thrombosis Forum (NATF)

Clot Care

Prefer not to answer

**Phase 2 – Selection of Education Topics and Methods**

**When you are in a hospital, how do you want to learn about blood clots? (check all that apply)**

On a piece of paper that you can read when you want

Talk with a doctor

Talk with a nurse

Talk with a pharmacist

Video on a TV screen

Video on a tablet (e.g. iPad)

Video on a smart phone (e.g. iPhone)

Interactive video game about blood clots

Internet

Other (please specify)

**When you are in a hospital, how would you like to learn about reducing your risk of getting a blood clot? (check all that apply)**

On a piece of paper that you can read when you want

Talk with a doctor

Talk with a nurse

Talk with a pharmacist

Video on a TV screen

Video on a tablet (e.g. iPad)

Video on a smart phone (e.g. iPhone)

Interactive video game about blood clots

Internet

Other (please specify)

**How would you like your family member or medical decision maker to learn about reducing your risk of getting a blood clot? (check all that apply)**

On a piece of paper that you can read when you want

Talk with a doctor

Talk with a nurse

Talk with a pharmacist

Video on a TV screen

Video on a tablet (e.g. iPad)

Video on a smart phone (e.g. iPhone)

Interactive video game about blood clots

Internet

Other (please specify)

**If you were given a paper handout about blood clots when you come to the hospital, would you read it?**

Yes

No

Other (please specify)

**If shots/injections and pills for preventing blood clots worked the same, which would you prefer?**

Shot/injection

Pill

No preference

**What information is most important to know about blood clots?**

Blood clots kill more people per year than AIDS, breast cancer, and car crashes combined

Blood clots are mostly preventable

Hospitalized patients are at higher risk for getting blood clots

Other (please specify)

**7. What other information is important to know about blood clots?**

**8. Is there anything else that you would like to add?**

**Phase 3 – Ranking of educational Topics and Methods**

**When you are in a hospital, how do you want to learn about blood clots? (select and rank three most preferred methods)**

*Video on a smart phone (e.g. iPhone)*

*Talk with a pharmacist*

*On a piece of paper that you can read when you want*

*Talk with a doctor*

*Video on a TV screen*

*Talk with a nurse*

*Video on a tablet (e.g. iPad)*

**If only a piece of paper was available, what information would you prefer? (Please rank in order of preference)**

*How to prevent blood clots*

*My risk for getting a blood clot*

*How to know when I might have a blood clot*

*What happens after I get a blood clot?*

**If only a piece of paper was available, I would be willing to read a: (Yes/No)**

1-page handout, front only

1-page handout, front and back

3-page handout

5-page handout

Scientific journal article

Reference list

Guideline from national clinical organizations

Other (please specify)

**If only a video was available, what is the maximum length that you would be willing to watch?**

5 minutes

10 minutes

15 minutes

20 minutes

Other (please specify)

**What type of video about prevention of blood clots would you like to see?**

Patients talking

Doctors/nurses/pharmacists talking

Both patients and doctors/nurses/pharmacists talking

Other (please specify)
